# Levothyroxine and the risk of adverse pregnancy outcomes in women with subclinical hypothyroidism: a systematic review and meta-analysis

**DOI:** 10.1186/s12902-021-00699-5

**Published:** 2021-02-27

**Authors:** Magnus Bein, Oriana Hoi Yun Yu, Sonia Marzia Grandi, Francesca Y. E. Frati, Ihab Kandil, Kristian B. Filion

**Affiliations:** 1 Department of Biology, McGill University, Montreal, Quebec Canada; 2 Department of Medicine, McGill University, Montreal, Quebec Canada; 3 Division of Endocrinology, Department of Medicine, Jewish General Hospital, Montreal, Quebec Canada; 4 Center for Clinical Epidemiology, Lady Davis Institute, Jewish General Hospital, Montreal, Quebec H3T 1E2 Canada; 5 Epidemiology Branch, Division of Intramural Population Health Research, Eunice Kennedy Shriver National Institute of Child Health and Human Development, National Institutes of Health, Bethesda, MD USA; 6 Department of McGill University Library & Archives, McGill University, Montreal, Quebec Canada; 7 Department of Epidemiology, Biostatistics, and Occupational Health, McGill University, Montreal, Quebec Canada

**Keywords:** Subclinical hypothyroidism, Levothyroxine, Pregnancy outcomes

## Abstract

**Background:**

Levothyroxine replacement therapy may decrease the risk of adverse pregnancy outcomes among women with subclinical hypothyroidism (SCH). The aim of this study is to conduct a systematic review and meta-analysis to examine the risk of adverse pregnancy, perinatal, and early childhood outcomes among women with SCH treated with levothyroxine.

**Methods:**

A systematic literature search was conducted using Ovid-Medline, Ovid-EMBASE, Pubmed (non-Medline), Ebsco-CINAHL Plus with full text and Cochrane Library databases. Randomized controlled studies (RCTs) and observational studies examining the association between treatment of SCH during pregnancy and our outcomes of interest were included. Studies that compared levothyroxine treatment versus no treatment were eligible for inclusion. Data from included studies were extracted and quality assessment was performed by two independent reviewers.

**Results:**

Seven RCTs and six observational studies met our inclusion criteria. A total of 7342 individuals were included in these studies. RCTs demonstrated several sources of bias, with lack of blinding of the participants or research personnel; only one study was fully blinded. In the observational studies, there was moderate to serious risk of bias due to lack of adjustment for certain confounding variables, participant selection, and selective reporting of results. Pooled analyses showed decreased risk of pregnancy loss (RR: 0.79; 95% CI: 0.67 to 0.93) and neonatal death (RR: 0.35; 95% CI: 0.17 to 0.72) associated with levothyroxine treatment during pregnancy among women with SCH. There were no associations between levothyroxine treatment and outcomes during labour and delivery, or cognitive status in children at 3 or 5 years of age.

**Conclusion:**

Treatment of SCH with levothyroxine during pregnancy is associated with decreased risks of pregnancy loss and neonatal death. Given the paucity of available data and heterogeneity of included studies, additional studies are needed to address the benefits of levothyroxine use among pregnant women with SCH.

**Supplementary Information:**

The online version contains supplementary material available at 10.1186/s12902-021-00699-5.

## Background

Subclinical hypothyroidism (SCH) is a common biochemical entity identified in women during pregnancy. SCH is diagnosed when the thyroid stimulating hormone (TSH) is elevated with a normal free thyroxine (FT4) level. Although most women with SCH are asymptomatic, previous studies have shown that SCH may be associated with adverse outcomes during pregnancy [1–3].

The thyroid hormone, FT4, is necessary for fetal growth and development. Insufficient thyroid hormone has been shown to impair fetal growth [4] and brain development [5] and it may have negative effects on neonatal survival [4]. Women with overt hypothyroidism during pregnancy require levothyroxine treatment [6]. However, there is uncertainty as to whether women with SCH during pregnancy should be treated as the benefits of treating SCH during pregnancy have not been consistently demonstrated [6–9].

Several studies have examined the association of SCH and adverse outcomes during pregnancy and long-term outcomes in mothers and children including pregnancy loss, pre-term delivery, gestational diabetes, gestational hypertension, eclampsia, placental abruption, low birth weight, and childhood cognitive outcomes [10–14]. Several of these studies reported increased risks of these outcomes among women with untreated SCH during pregnancy [2, 3, 14]. However, there was heterogeneity between studies with respect to the timing of initiation of levothyroxine, the study population, the underlying cause of SCH, and the estimated treatment effects [1, 2, 15, 16]. As a result of the discordant findings, the 2017 American Thyroid Association (ATA) guidelines recommended levothyroxine therapy for women with SCH (defined as a TSH level greater than the pregnancy-specific range) and thyroid autoimmune disease (defined as the presence of anti-thyroid peroxidase antibodies [TPOAb)]). For women with negative TPOAb levels, the guidelines recommended treatment with levothyroxine therapy for TSH levels greater than 10mIU/L. [9] However, levothyroxine therapy was not recommended for women with no antibodies and a TSH within the pregnancy-specific reference range.

Previous meta-analyses have been performed on this topic. However, these meta-analyses have focused on comparing women with SCH with euthyroid women during pregnancy [10, 12] or only included randomized controlled trials (RCTs), which are very few to this date [16]. To our knowledge, only two meta-analyses have assessed the effects of levothyroxine treatment among women with SCH during pregnancy. The meta-analysis by Rao et al. (2019) [17], pooled studies including women with SCH and euthyroid women with thyroid autoimmune disease and compared women treated with levothyroxine versus no treatment. The meta-analysis by Nazapour et al. (2019), [18], compared women with SCH during pregnancy treated with levothyroxine with women who were euthyroid. However, this meta-analysis performed a subgroup analysis comparing the risk of pregnancy loss associated with levothyroxine treatment versus no treatment among women with SCH and found that levothyroxine was associated with a decreased risk of pregnancy loss among SCH women treated with levothyroxine during pregnancy (odds ratio: 0.78; 95% confidence interval [CI]: 0.66–0.94) [18]. Although women with SCH treated with levothyroxine have normal TSH levels similar to euthyroid women, it is uncertain whether euthyroid women are comparable to women with treated SCH.

Due to the lack of good quality evidence for treatment of SCH during pregnancy, it is unclear whether levothyroxine treatment should be given to women with isolated SCH during pregnancy [9]. To examine this question, we conducted a systematic review and meta-analysis of studies comparing the risk of maternal and fetal outcomes in women with SCH who were treated or not treated with levothyroxine during pregnancy.

## Methods

Our systematic review was conducted following a pre-specified protocol and is reported based on the guidelines outlined in the Preferred Reporting Items for Systematic Reviews and Meta-Analyses (PRISMA) statement [19]. The study protocol is available upon request from the corresponding author. The inclusion criteria and analyses described below do not deviate materially from those specified in this protocol.

### Literature search

We systematically searched Ovid MEDLINE (Appendix 1), Ovid EMBASE (Appendix 2), Ebsco-CINAHL Plus with Full Text (Appendix 3), Pubmed (for articles not indexed in Medline) (Appendix 4), and Cochrane Library from inception to July 18, 2018 to identify studies that examined the association between treatment of SCH during pregnancy and adverse pregnancy outcomes. A medical librarian (FF) designed and conducted the searches (see Appendix for full search strategies). The Ovid-Medline search was peer reviewed by a second librarian using the Peer Review of Electronic Search Strategies (PRESS) guideline [20]. No language restrictions were applied. We also scanned the references of relevant articles, searched for citing articles (snowballing), and conducted a search of the grey literature to retrieve studies not identified by our primary search. We reviewed previous systematic reviews and meta-analyses of levothyroxine treatment among women with SCH during pregnancy and retrieved studies not identified in our search.

### Study selection

We included interventional and observational studies that reported the risk of adverse pregnancy outcomes with and without levothyroxine treatment among women with SCH. Inclusion was restricted to studies in which SCH was defined by a TSH level between 2.5 and 10 mIU/L at any time during pregnancy and included women with SCH identified pre-pregnancy. We based our definition of SCH on the 2011 ATA guidelines [21], which is a broader definition compared to that included in the more recent 2017 ATA guidelines which recommends treatment for SCH during pregnancy depending on the presence of thyroid autoimmune disease [9]. Furthermore, given that the ATA guidelines on management of SCH during pregnancy are similar for women with pre-existing and newly diagnosed SCH, studies that included women with pre-existing SCH and were initiated on levothyroxine treatment pre-conception were included. The use of this broader definition allowed for the inclusion of all available evidence. Studies were required to report at least one of the following outcomes: pregnancy loss (spontaneous abortion and stillbirths), spontaneous abortion (pregnancy loss before 20 weeks of gestation), and stillbirth (death of an infant occurring after 20 weeks of gestation). We also included the assessment of other outcomes reported in the studies selected, including intrauterine growth restriction, preterm delivery, preterm labor, low Apgar score (< 7 at 5 min after birth), low birth weight, or behavioural and cognitive development of the child. We excluded uncontrolled studies, systematic reviews and meta-analyses, cross-sectional studies, letters to the editor and commentaries, and animal studies. Finally, we excluded conference abstracts as they contain insufficient information for quality assessment.

After removing duplicates, two independent reviewers screened the titles and abstracts of identified publications, with any publication deemed potentially relevant by either reviewer carried forward to full-text review. Discrepancies during full-text review between reviewers were resolved by consensus or a third reviewer (IK or KBF).

### Quality assessment and data extraction

Quality assessment and data extraction were performed for all included studies by two independent reviewers (MB and OY), with disagreements resolved by consensus or by a third reviewer (IK or KBF). The Cochrane Risk of Bias tool was used for RCTs [22] and the Risk of Bias In Non-randomised Studies of Interventions (ROBINS)-I tool was used for observational studies [23]. We extracted information on study design, study population characteristics (size, demographics, location, study period), SCH definition, key findings, and frequencies and effect estimates and 95% CIs for the association between levothyroxine and adverse pregnancy outcomes. For observational studies, adjusted effect estimates were extracted.

### Statistical analysis

Given the paucity of RCTs that assessed the risk of adverse events during pregnancy with levothyroxine treatment among women with subclinical hypothyroidism, we performed our primary analysis, pooling findings from RCTs and observational studies [24]. By including the totality of evidence in this area of research, we increase precision of our estimates [24]. We also performed a secondary analysis whereby we used meta-regression to study the effects of study design (i.e. RCTs and observational studies) on the association between levothyroxine treatment and pregnancy loss and other adverse outcomes among women with SCH. For each binary outcome, we pooled risk ratios (RR) using Dersimonian and Laird random-effects models with inverse variance weighting [25], applying the Jackson and Knapp-Hartung extensions [26, 27]. A continuity correction of 0.5 was used for both the treatment and reference groups when a frequency of zero was present. For continuous outcomes, we estimated the weighted mean difference using a similar approach [28]. Heterogeneity was assessed by the tau-squared estimators, and the I^2^ statistics. We conducted the analyses using the *meta* package [29] in *R* [30].

### Sensitivity analyses

We performed six sensitivity analyses. First, to address the influence of levothyroxine treatment for women with SCH caused by an autoimmune condition, we repeated our analyses including only studies that addressed the risk of adverse pregnancy outcomes among women with TPOAb positivity who were treated with levothyroxine versus untreated women. Second, the meta-analysis was repeated after excluding studies that assessed women with a history of infertility or recurrent miscarriages, as women with these conditions may have a higher risk of adverse pregnancy outcomes. Third, given that one of the prospective clinical trials did not utilize randomization compared to the other prospective clinical studies [31], we repeated our primary meta-analysis excluding this study. Fourth, we performed stratified analyses based on the quality of RCTs and observational studies that assessed the association between levothyroxine use and the risk of pregnancy loss among women with SCH during pregnancy. Fifth, we conducted an influence analysis in assessing the association between levothyroxine treatment and pregnancy loss to determine if any study had a significant impact on our results. Finally, we repeated our meta-analyses using a continuity correction of 0.1 for both the treatment and reference groups.

## Results

**Fig. 1:**
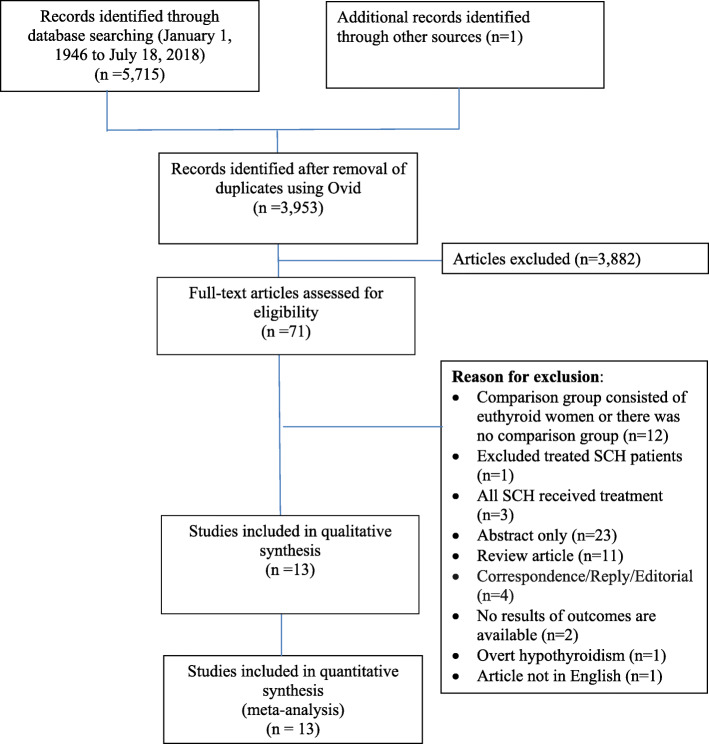
Flowchart diagram (Figure adapted from PRISMA 2009 Flow Diagram) [19]

Seven RCTs and six cohort studies were included from a total of 3953 articles identified by our search (Fig. 1). Thyroid hormone levels of subjects included in the studies were consistent in terms of the definition of SCH, with some minor heterogeneity for the specific populations the subjects were drawn from. For RCTs and observational studies, the intervention was initiation of levothyroxine for women identified with SCH. The timing of initiation of treatment, the dosing, and adjustment of treatment varied widely across studies (Table 1).

**Table 1:** Summary of studies included in the systematic review and meta-analysis

Study and research institution	Study design and Size (N)	Geographic Region and Period	Serum TSH and fT4 for SCH diagnosis	Gestational age levothyroxine started with initial dose and adjustment	Outcomes	Findings
Bernardi et al.; 2013 [32] University of Chicago	Cohort study *N* = 39	United States July 2004–December 2011	TSH > 2.5mIU/L with normal fT4 level (0.9–1.7 ng/dL) measured before conception	Initiated if SCH was identified pre-conception Initial dose and adjustment unknown.	Live-birth rates	Among women with a history of > 2 pregnancy losses and SCH, women who received treatment for SCH did not have an increased live birth rate compared to women who did not receive levothyroxine treatment (per-pregnancy live birth rate: SCH treated: 48% (22/46) vs. SCH untreated: 52% (12/23).
Maraka et al. 2016 [33] Mayo Clinic	Retrospective Cohort *N* = 366	Rochester, Minnesota January 2011–December 2013	TSH 2.5–10 mIU/L in first trimester & 3–10 mIU/L in other trimesters normal fT4 (> 0.8 ng/dL)	Started at median GA 9.1 weeks	Pregnancy loss defined as miscarriage and stillbirth, preterm delivery (< 37 weeks), premature rupture of membranes, placental abruption, gestational diabetes, gestational hypertension, pre-eclampsia, eclampsia, intrauterine growth restriction, birth weight, Apgar score at 5 min, admission to the neonatal intensive care unit, neonatal death (during immediate postpartum period until discharge of the mother) and duration of hospital stay.	Treated pregnancies had a lower birth weights and no Apgar score less than 7. Other pregnancy outcomes were statistically similar between groups. Pregnancy loss OR: 2.44; 95% CI: 0.80–8.87 Preterm delivery OR: 3.06; 95% CI: 0.96–12.28 Gestational diabetes OR: 3.31; 95% CI: 0.91–16.57 Gestational hypertension OR: 0.64; 95% CI: 0.23–1.93 Pre-eclampsia OR: 3.37; 95% CI: 0.66–26.84 Premature rupture of membranes OR: 0.71; 95% CI: 0.29–1.79 Intrauterine growth restriction OR: 1.45; 95% CI: 0.23–28.1 Placenta previa and placental abruption: not enough events to do multivariate analyses NICU admission OR: 1.94; 95% CI: 0.38–15.36 Birth weight < 2500 g OR: 16.4; 95% CI: 2.7–326.9 Neonatal death and congenital malformations: not enough events to do multivariate analyses
Al-Anbari, 2017	Prospective study N=149	High Institute of Infertility diagnosis and assisted reproductive technologies/Al-Nahrain University, Iraq	TSH > 2.5mIU/L prior to conception	Initiated if SCH was identified pre-conception	Pregnancy rate, miscarriage rate, multiple pregnancy rate and live birth rate	Significantly increased pregnancy rate (19/75 among levothyroxine treated versus 8/74 among women not given treatment). No multiple pregnancies in both groups. No difference in miscarriage rate (2/75 among levothyroxine treated versus vs 2/74 among women not given treatment).
Maraka et al. 2017 [34] OptumLabs Data Warehouse	Retrospective Cohort *N* = 5405	United States January 2010–December 2014	TSH 2.5–10 mIU/L from 1 month prior to 3 months after first prenatal visit fT4 0.8 ng/dL or total thyroxine 7.5 mcg/dL	Started at median GA 28.7 weeks before birth Unknown initial dose. Median dose: 50 mcg daily (range 25–300mcg daily)	Pregnancy loss defined as miscarriage and still-birth, preterm delivery, preterm labor, premature rupture of membranes, placental abruption, gestational diabetes, gestational hypertension, pre-eclampsia, poor fetal growth, tachycardia	Treatment of SCH was associated with decreased risk of pregnancy loss but was associated with increased risk of other adverse pregnancy related outcomes. Pregnancy loss OR: 0.62; 95% CI: 0.48–0.82 Preterm delivery OR: 1.6; 95% CI: 1.14–2.24 Preterm labor OR: 1.14; 95% CI: 0.89–1.46 Premature rupture of membranes OR: 0.97; 95% CI: 0.66–1.42 Placental abruption OR: 1.60; 95% CI: 0.65–3.93 Gestational diabetes OR: 1.37; 95% CI: 1.05–1.79 Gestational hypertension OR: 1.27; 95% CI: 0.88–1.82 Pre-eclampsia OR: 1.61; 95% CI: 1.10–2.37 Poor fetal growth OR: 1.12; 95% CI: 0.84–1.5 Tachycardia OR: 1.77; 95% CI: 1–3.11
Nazarpour et al. 2017 [35] Shahid Beheshti Medical University	RCT *N* = 1294	Tehran, Iran September 2013–February 2016	TSH 2.5–10 mIU/L fT4 1–4.5 TPOAb positive	Started 4 to 8 d after prenatal visit, the mean GA at initial visit was 10.8 weeks. 1 mcg/kg daily Dose adjustment not described.	Preterm delivery, neonatal admission, placental abruption, still birth, GA mean birth weight, neonate height, birth head circumference, neonatal TSH	There were no significant differences in preterm delivery or neonatal admission between treated and untreated women with SCH (TSH < 4mIU/L) treated versus untreated but there were significant differences in preterm delivery and neonatal admission between treated and untreated women with TSH > 4mIU/L. There was also a significant decrease in neonatal admission among women treated for SCH versus women who did not receive treatment [number (%): 2 (3.6) vs.12 (20.7)] There were no significant differences in placental abruption, still birth, and gestational age between treated versus untreated women with SCH. There were no significant differences in mean birth weight, head circumference and neonate TSH levels between study groups.
Nazarpour et al. 2018 [36] Shahid Beheshti Medical University	RCT *N* = 354	Tehran, Iran September 2013–February 2016	TSH 2.5–10 mIU/L fT4 1–4.5 TPOAb negative	Started 4–8 days after first prenatal visit, which was at 11.2–12.2 weeks of gestation Dosed at 1 mcg/kg daily. Dose adjustment not described	Preterm delivery, placental abruption, stillbirth, neonatal admission, birth weight, mean gestational age neonate height, birth head circumference neonatal TSH	Significant difference in pre-term delivery when TSH > 4 mIU/L and treated with levothyroxine versus no treatment, RR: 0.38; 95% CI: 0.15–0.98. There was no significant difference in the risk of other adverse pregnancy outcomes among women with SCH treated versus untreated. Number (%) of outcomes treated versus untreated: Preterm delivery: 18 (9.8) vs. 21 (11.5) Neonatal admission: 8 (4.5) vs. 9 (4.9) Placental abruption: 3 (1.6) vs. 0 Stillbirth: 0 vs. 0 Gestational age: 38.03 (1.4) vs. 37.9 (1.5) Mean (standard deviation) Birth weight: 3190.82 g (455.13) vs. 3203.1 g (497.1) Neonate height: 50.1 cm (2.3) vs. 50.2 cm (2.7) Birth head circumference: 34.6 cm (1.4) vs. 34.7 cm (1.6) Neonatal TSH (mIU/L): 1.1 (0.5–1.9) vs. 1.1 (0.5–2.1)
Casey et al. 2017 [37]	RCT *N* = 677 women with SCH, 526 with hypothyroxinemia	United States October 2006–October 2009	TSH > 3mIU/L or fT4 < 0.86 ng/dl with TSH between 0.08 and 3.99mIU/L	Started at GA 10–24 weeks; average GA 16.6 +/− 3 week standard deviation Initial dose 100 mcg daily. Monthly adjustment to maintain TSH 0.1–2.5 mIU/L Max dose: 200 mcg daily Treatment group normalized TSH by median of GA 21 weeks	Multiple pregnancy and neonatal outcomes. For maternal outcomes: preterm birth (< 34 and < 37 weeks), placental abruption, gestational hypertension, preeclampsia, gestational diabetes. For fetal and neonatal outcomes: stillbirth/miscarriage, neonatal death, Apgar score at 1 and 5 min, birth weight (< 10 percentile), head circumference, respiratory distress syndrome, necrotizing enteritis, bronchopulmonary dysplasia, respiratory therapy > 1 day, number of days in nursery. Annual cognitive testing over first 5 years, IQ at 5 yo. or general conceptual ability at 3 yo	No differences in adverse pregnancy and neonatal outcomes between levothyroxine treated versus placebo group. No differences in IQ score at the age of 5 years or death at the age of < 3 years between treated and untreated SCH (median IQ score for children in levothyroxine treated group: 97 (95% CI: 94–99) versus 94 (95% CI: 91–95) in placebo.
Lazarus et al. 2012 [38]	RCT *N* = 794	Wales & Cardiff, UK Ospendale Sant’Anna, Turin, Italy Period of time not mentioned but followed children at age 3 years	TSH > 97.5th percentile and f T4 < 2.5th percentile	Started at GA 12–13 weeks Initial dose of 150 mcg daily. Adjustment 6 weeks after beginning treatment and at 30 weeks GA to target TSH 0.1–1 mIU/L	IQ at 3 years of age	No significant differences in IQ scores at age 3 years between children born of mothers with SCH treated versus placebo during pregnancy: IQ score treated: 99.2 +/− 13.3 vs. untreated: 100 +/− 13.3.
Kim et al. 2011 [39]	RCT *N* = 64	Seoul, South Korea March 2006–September 2009	TSH > 4.5mIU/L with normal fT4	Started prior to IVF/ICSI treatment (before pregnancy). Initial dose 50 mcg daily. Adjustment: First trimester titration to maintain TSH < 2.5 mIU/L to max dose 100mcg daily (12/17) and 125mcg daily (1/17)	Embryo implantation rate, total amount and days of rhFSH administered, number of retrieved, mature, fertilized oocytes, and good quality embryos, clinical pregnancy rate per cycle, and miscarriage rate, preterm birth (< 34 weeks) and live birth (delivery of fetus> 20 weeks with signs of life)	Significant increase in embyro quality and implantation when women with SCH were treated versus untreated. Significant decrease in miscarriage and increase in live birth rate per cycle when women with SCH were treated versus untreated. Miscarriage rate in treated versus untreated: 0/17 vs. 4/12 Live birth rate per cycle in treated versus untreated: 17/32 vs. 8/32
Ju et al. 2016 [40] Beijing Friendship Hospital of Capital Medical University	Prospective cohort *N* = 457	Beijing, China October 2010–September 2013	TSH > 97.5th percentile and fT4 2.5th–97.5th percentile	Started at approx. GA 10 weeks Initial dose 100 mcg daily. Adjusted by 100 mcg to maintain TSH 2.5–97.5 percentile.	Premature rupture of membranes, fetal macrosomia, gestational diabetes, hypertensive disorders in pregnancy, postpartum hemorrhage, preterm labor, oligohydramnios, fetal distress, and low birth weight	Overall risk of pregnancy complications in control group significantly higher than in treated group (OR: 1.219; 95% CI 1.139–1.304). For single outcomes, there was a statistically increased risk of gestational diabetes among untreated versus treated women with SCH (OR: 1.938; 95% CI: 1.267–2.964) and fetal macrosomia (OR:3.081; 95% CI: 1.783–5.326).
Wang et al. 2012 [41] Liaoning Provincial Key Laboratory of Endocrine Diseases	Prospective cohort *N* = 196	Shenyang, China 2007–2009	TSH > 2.5 mIU/L with a fT4 between 12 pmol/L and 23.34 pmol/L in the first 12 weeks of pregnancy	Started at approximately 6 weeks. Initial dose at 50 μg, 75 μg, or 100 μg daily Adjustment every 4 weeks by serum TSH (mIU/L): 2.5–5: 50 μg daily 5–8: 70 μg daily > 8: 100 μg daily	Spontaneous abortion, anemia, hypertension, premature delivery, low birth weight, post-partum hemorrhage, Apgar score ≤ 7 at 5 min	Levothyroxine treatment in women with SCH decreased the incidence of spontaneous abortions compared to no treatment but this was not statistically significant. Outcomes in treated versus untreated women with SCH: Spontaneous abortions: 2 (7.14%) vs. 26 (15.47%) Anemia: 3 (10.71%) vs. 35 (20.83%) Hypertension: 1 (3.57%) vs. 2 (1.19%) Premature delivery: 0 vs. 9 (5.36%) Low birth weight: 0 vs. 4 (2.38%) Hemorrhage: 0 vs. 2 (1.19%) Apgar score < 7 at 5 min: 0 vs. 4 (2.38%)
Zhao et al. 2018 [42] Shuguang Hospital Affiliated to Shanghai University of Traditional Chinese Medicine	RCT *N* = 93	Shanghai, China January 2014–October 2016	T1:TSH > 2.5mIU/L T2: TSH > 3mIU/L	Started either in T1 at 8-10 weeks or T2 at 13–16 weeks. Initial dose 25mcg daily. Max dose: 100 mcg daily	Gestational hypertension, anemia, gestational diabetes, pre-eclampsia, premature labor, pregnancy loss, post-partum hemorrhage, low birth weight (< 2500 g)	Pregnancy complications: no significant different between treated versus untreated groups for individual outcomes. However, for combined outcomes: treatment given during T1 had significantly less complications than women who were not treated (Number of total adverse pregnancy outcomes among women treated at T1: 1/31 vs. 10/31 (treated at T2) vs. 12/31 (no treatment).
Zhang et al. 2017 [12]	Retrospective cohort N = 9	ZhongDa, China January 2014–May 2014	TSH 0.27–4.2 mIU/L and fT4 0.93–1.70 ng/dL (in T2)	Started in second trimester. Dose at 50 mcg daily	Premature delivery (< 37 weeks), Apgar score, birth weight	No significant differences in the rate of premature delivery (0/1 in treated vs. 3/8 untreated), Apgar (10 +/− 0 in treated vs. 9.8+/− 0.61 untreated), and birth weight (3.67+/− 0.6 kg in treated vs. 3.48+/− 0.54 kg in untreated) in women with treated and untreated SCH.

Abbreviations: *CI* confidence intervals; *C/S* cesarean section; *ER* emergency room; *fT4* free thyroxine; *GA* gestational age; *IQ* intelligence quotient; *IVF* in vitro fertilization, *NICU* neonatal intensive care unit; *OR* odds ratio; *RCT* randomized controlled trial; *rhFSH* recombinant human follicular stimulating hormone; *SCH* subclinical hypothyroidism; *T1* first trimester; *T2* second trimester; *TSH* thyroid stimulating hormone; *yo* years old

### Quality assessment

#### RCTs

One RCT fulfilled all quality criteria [37] and was classified as high-quality study and the remaining six RCTs were of moderate quality (Table S1). Five studies did not meet the criteria in at least three domains of bias [31, 35, 38, 39, 42] and one study was deficient in two criteria [36]. Six of the included RCTs were randomized [35–39, 42], however half did not perform appropriate allocation concealment [38, 39, 42]. In all except one study [37], participants and personnel were not blinded [31, 35, 36, 38, 39, 42]. Assessors of the outcomes were blinded in four of the seven RCTs [35–38]. Reporting bias was a concern in two RCTs due to selective reporting of subgroup analyses [35, 36].

#### Observational studies

The included cohort studies had a moderate to serious risk of bias (Table S2). Three domains of bias drove this overall quality assessment: confounding, participant selection, and selective reporting of results. The lack of control for important confounding variables was moderate to critical in all studies. The risks of selection bias, and bias due to selective reporting of results were moderate in three of six studies [33, 34, 41], with one at serious risk [40]. Risk of bias from measurement of outcomes was low to moderate. Interventional risks and missing data biases were low, except in one study [12].

#### Fetal outcomes

**Fig. 2:**
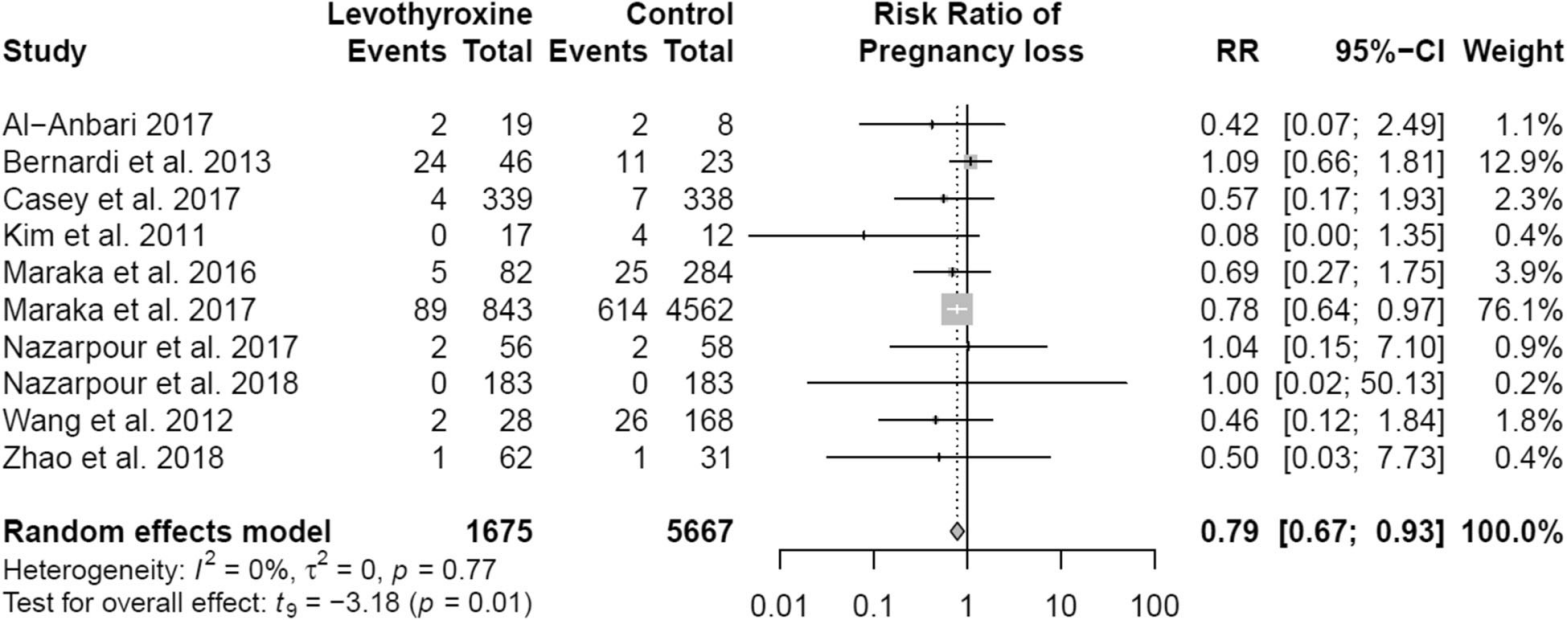
Random-effects meta-analysis of pregnancy loss associated with levothyroxine treatment versus no treatment among women with subclinical hypothyroidism during pregnancy presented as a risk ratio (RR) and 95% confidence interval (CI)

**Fig. 3:**
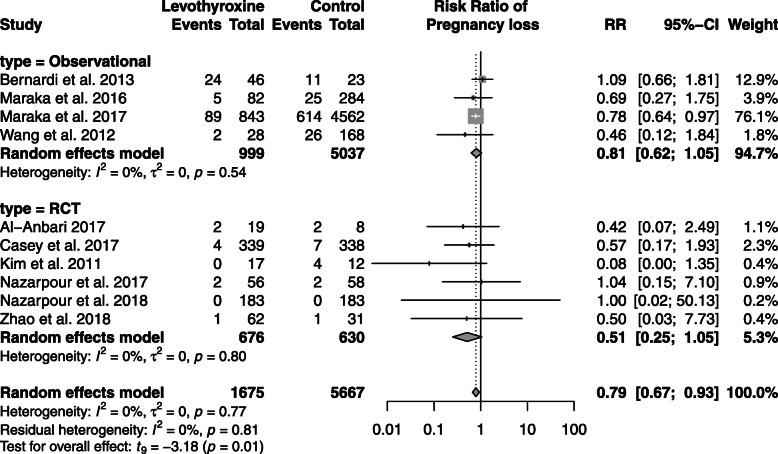
Random-effects meta-analysis of pregnancy loss associated with levothyroxine treatment versus no treatment among women with subclinical hypothyroidism during pregnancy stratified by type of study (randomized clinical trials [RCT] and observational studies) presented as a risk ratio (RR) and 95% confidence interval (CI)

Congenital malformation, fetal distress, fetal macrosomia, oligohydramnios, placenta previa were reported in single studies and thus meta-analyses were not possible (Table 2). Meta-analyses were performed for the following outcomes: intrauterine growth restriction (2 studies), spontaneous abortion (4 studies), and pregnancy loss (10 studies) (Table 2). Compared with non-use, levothyroxine treatment among women with SCH was associated with a decreased risk of pregnancy loss (RR: 0.79; 95% CI: 0.67–0.95) (Fig. 2). When we stratified the meta-analyses by study design, there was a suggestion of greater benefits with respect to pregnancy loss in RCTs (RR: 0.51; 95% CI: 0.25–1.05) than observational studies (RR: 0.81; 95% CI: 0.62–1.05) but 95% CIs overlapped between the two (Fig. 3). We did not observe an association between the use of levothyroxine among women with SCH during pregnancy and the risk of intrauterine growth restriction and spontaneous abortion (Table 2, Table 3 and Figures S1-S2).

**Table 2:** Summary of binary outcomes between pregnant women with subclinical hypothyroidism treated with and without levothyroxine, expressed risk ratio (RR) with 95% confidence intervals (CI)

Outcomes	No. of studies	Pooled sample	Risk ratio (95% CI)^*^
***Fetal***			
Congenital malformation	1	336	0.37 (0.02 to 6.8)
Fetal distress	1	457	0.78 (0.59 to 1.0)
Fetal macrosomia	1	457	0.32 (0.19 to 0.56)
Intrauterine growth restriction	2	5771	1.06 (0.59 to 1.90)
Oligohydramnios	1	457	1.48 (0.60 to 3.7)
Placenta abruption	5	6928	0.98 (0.37 to 2.61)
Placenta previa	1	366	0.49 (0.03 to 9.5)
Spontaneous abortion	4	366	0.45 (0.13 to 1.56)
Pregnancy loss (still births and spontaneous abortions)	10	7342	0.79 (0.67 to 0.93)
***Perinatal***			
Postpartum hemorrhage	3	746	0.88 (0.18 to 4.22)
Premature rupture of membranes	3	6228	0.94 (0.52 to 1.70)
Preterm delivery	7	7217	0.77 (0.47 to 1.25)
Preterm labour	2	5862	1.05 (0.56 to 1.97)
Still birth	4	536	NA
***Neonatal***			
APGAR under 7 at five minutes	3	1209	0.42 (0.03 to 5.31)
Low birth weight	5	1759	0.80 (0.26 to 2.43)
Neonatal death	2	1013	0.35 (0.17 to 0.72)

^*^When only one study has the reported response, the data summarized are taken from the single study

*NA* No stillbirths were observed in control or treatment groups in any studies reporting this outcome

**Table 3:** Summary of binary outcomes between pregnant women with subclinical hypothyroidism treated with and without levothyroxine in randomized controlled trials and observational studies, expressed as risk ratios (RR) with 95% confidence intervals (CI)

	Observational studies		Randomized controlled trials	
Outcomes	No. of studies	Risk ratio (95% CI)^*^	No. of studies	Risk ratio (95% CI)
***Fetal***				
Intrauterine growth restriction	2	1.06 (0.59 to 1.90)	0	NA
Placenta abruption	2	1.06 (0.81 to 1.38)	3	0.93 (0.01 to 101.99)
Spontaneous abortion	1	0.46 (0.12 to 1.84)	3	0.44 (0.03 to 6.83)
Pregnancy loss (still births and spontaneous abortions)	4	0.81 (0.62 to 1.05)	6	0.51 (0.25 to 1.05)
***Perinatal***				
Postpartum hemorrhage	2	1.16 (1.06 to 1.27)	1	0.33 (0.06 to 1.89)
Premature rupture of membranes	3	0.94 (0.52 to 1.70)	0	NA
Preterm delivery	3	0.82 (0.13 to 4.98)	4	0.72 (0.39 to 1.35)
Preterm labour	2	1.05 (0.56 to 1.97)	0	NA
Still birth	4	0.75 (0.39 to 1.42)	0	NA
***Neonatal***				
APGAR under 7 at five minutes	2	0.24 (0.00 to 68,937.70)*	1	0.66 (0.11 to 3.95)
Low birth weight	3	0.55 (0.03 to 11.63)	2	0.86 (0.00 to 3722.22)
Neonatal death	1	0.37 (0.02 to 6.83)	1	0.33 (0.01 to 8.13)

NA –non-applicable

*There were no events in the levothyroxine exposed groups

#### Perinatal outcomes

Levothyroxine treatment among women with SCH was not associated with placenta abruption, postpartum hemorrhage, premature rupture of membranes, preterm delivery, or preterm labour (Table 2, Table 3 and Figures S3-S7). Four studies examined stillbirth [31, 35, 36, 39] but no stillbirths occurred among study participants.

#### Neonatal outcomes

Neonatal outcomes assessed included neonatal death, 5-min Apgar score, and low birthweight (Table 2, Table 3). Levothyroxine treatment compared to no treatment among women with SCH was associated with a decreased risk of neonatal death (RR 0.35; 95% CI: 0.17–0.72) (Figure S8). Levothyroxine treatment was not associated with the risk of low 5-min Apgar score (Figure S9) and low birthweight (Figure S10). Additionally, levothyroxine treatment was not associated with head circumference in the two RCTs that reported this outcome (Table 4).

**Table 4:** Summary of mean difference with 95% confidence intervals (CI) between neonatal head-circumference and pediatric cognitive outcomes in pregnant women with subclinical hypothyroidism treated with and without levothyroxine

Outcomes	Unit of measure	No. of Studies	Pooled Sample	Mean Difference (95% CI)
***Neonatal***				
Neonatal head circumference (at birth)	cm	2	1031	−0.042 (−0.67 to 0.58)
***Pediatric***				
Child Behaviour Checklist T-score at 3 years old	percentile	2	1409	−0.50 (−4.5 to 3.5)
IQ at 3 to 5 years old	percentile	2	1443	0.94 (−23 to 25)

#### Cognitive outcomes in children at 3 to 5 years of age

Only two RCTs assessed the association between levothyroxine treatment and behavioral and cognitive outcomes of children of women with SCH (Table 4). These RCTs showed that treatment with levothyroxine in women with SCH was not associated with behavioural or cognitive performance in the children at the age of 3 to 5 years [37, 38].

#### Sensitivity analyses

Two studies addressed whether levothyroxine treatment was associated with pregnancy outcomes among women with SCH caused by autoimmune disease (diagnosed by elevated TPOAb levels) [35]. Nazarpour et al. [35, 42] conducted a single-blinded RCT of pregnant women with SCH and elevated TPOAb. They found a decreased risk of preterm delivery associated with levothyroxine treatment compared to no treatment (7.1% versus 23.7%) [35]. Zhao et al. [42] randomized 93 pregnant women with SCH to treatment with levothyroxine or no treatment during the first and second trimesters of pregnancy. Although this study identified women with elevated TPOAb levels, the investigators did not compare the effect of treatment versus not on adverse pregnancy outcomes among women with SCH and elevated TPOAb levels. As such, a meta-analysis of the findings from these two studies was not possible.

Sensitivity analyses that excluded studies of women with a history of infertility or who conceived with fertility treatments [31, 39] and women with recurrent miscarriages [32] were consistent with those of our primary analysis (Figure S11). Furthermore, the results from the primary analysis remained consistent after excluding the study by Al-Anbari [31] (Figure S12). Given that there was no distribution in the quality of the RCTs, a stratified analysis on the quality of RCT studies was not feasible. When stratifying observational studies based on quality, there is a non-statistically, significantly decreased risk of pregnancy loss associated with levothyroxine treatment among women with SCH in studies considered at high risk and moderate risk of bias (Figure S13). In the influence analysis, the results remained consistent, showing an association between levothyroxine treatment versus no treatment was associated with a decreased risk of pregnancy loss. However, this association was no longer significant when the study by Maraka et al. [34] was excluded. Finally, our findings remain consistent when a continuity correction of 0.1 was used in our meta-analyses (Table S3).

## Discussion

In this systematic review and meta-analysis, we assessed the available evidence regarding the use of levothyroxine in treating SCH during pregnancy. We found that the use of levothyroxine among women with SCH was associated with a decreased risk of pregnancy loss and neonatal death relative to non-use. Although available data are limited, there is also evidence that levothyroxine treatment is associated with improved fetal outcomes, including reductions in fetal distress and macrosomia. We did not observe associations between treatment with levothyroxine and other adverse outcomes during pregnancy, labor and delivery and postpartum. Finally, there was no evidence of associations between levothyroxine use during pregnancy and cognitive outcomes in children. However, there was heterogeneity with respect to study populations and timing of initiation of levothyroxine between the included studies. Given the limited quality of the available data and its heterogeneity, additional high-quality studies are needed.

There is well-established evidence for the need to treat women with overt hypothyroidism during pregnancy as studies have shown that untreated hypothyroidism during pregnancy leads to increased risk of pregnancy complications, including increased risks of preterm birth, low birth weight, and stillbirth [43, 44]. Throughout gestation, the fetus is dependent on the mother’s supply of thyroid hormone, [45, 46] even after the fetal thyroid gland begins to function at approximately 12 weeks gestation. Thyroid hormone is a key moderator of fetal neurological development, fetal growth, and development of somatic tissue [8, 15]. As an adaptation to increased demand, physiological changes in the mother result in elevated maternal serum thyroxine concentration throughout pregnancy [8, 15, 46]. Furthermore, given that thyroid hormone plays an important role in brain development, untreated hypothyroidism during pregnancy may lead to deficiencies in fetal neurocognitive development and lower intelligence quotient (IQ) in the offspring [47, 48]. Thus, based on the known essential role of thyroid hormone during pregnancy, it is possible that untreated SCH can lead to adverse outcomes during pregnancy including an increase in pregnancy loss [49].

A recent systematic review and meta-analysis of studies comparing women with SCH and euthyroid women during pregnancy found that SCH was associated with an increased risk of multiple adverse maternal and fetal outcomes [10], including pregnancy loss (RR: 2.01; 95% CI: 1.66–2.44), placental abruption (RR: 2.14; 95% CI: 1.23–3.70), premature rupture of membranes (PROM) (RR: 1.43; 95% CI: 1.04–1.95) and neonatal death (RR: 2.58; 95% CI: 1.41–4.73). A recent systematic review and meta-analysis of RCTs assessed whether levothyroxine treatment during pregnancy among women with SCH has an impact on obstetrical and fetal outcomes [16]. This study included 3 trials and found no difference in obstetrical and neonatal outcomes, including childhood IQ and neurocognitive outcomes among children born to women with SCH who were treated with levothyroxine compared to those who received no treatment [16]. Thus, evidence from RCTs suggests that there is no significant reduction in adverse fetal outcomes associated with levothyroxine treatment in women with SCH. Finally, a meta-analysis by Rao et al. [17] showed that levothyroxine treatment among women with SCH and women with thyroid autoimmune disease was associated with a decreased risk of pregnancy loss and preterm birth compared to women who received no treatment. In a sub-group analysis of women with SCH, levothyroxine treatment was associated with a decreased risk of pregnancy loss compared to no treatment (RR: 0.43; 95% CI: 0.26–0.72) but there was no association between levothyroxine treatment and preterm birth (RR: 0.67; 95% CI: 0.41–1.12). Although this meta-analysis included fewer studies than our meta-analysis, [17] the findings are consistent with the current study. Nazarpour et al. [18] performed a meta-analysis comparing women with SCH during pregnancy treated with levothyroxine with women who were not treated or were euthyroid. In a subgroup analysis, they compared women with SCH who were treated with levothyroxine versus no treatment, and found a decreased risk of pregnancy loss associated with levothyroxine treatment (odds ratio: 0.78; 95% CI: 0.66–0.94). Although the types of studies included in this meta-analysis differed from ours and included studies that had different TSH targets for treatment of SCH (i.e. targeted TSH to < 4.2 mIU/L [50]), the findings are consistent with our study. In addition, this previous meta-analysis did not assess neonatal and cognitive outcomes in children.

The majority of the studies performed to date initiated levothyroxine during the first trimester with only two studies addressing the effects of initiating levothyroxine at other times during pregnancy [40]. Ju et al. [40, 42] found that initiation of levothyroxine during the first trimester decreased the risk of PROM, gestational diabetes, postpartum hemorrhage, gestational hypertension, and fetal macrosomia compared to women who received levothyroxine treatment in the second and third trimester [40]. Zhao et al. [42] also showed that initiation of levothyroxine during the first trimester was associated with a decreased risk of adverse pregnancy outcomes (i.e. composed of premature labor, pregnancy loss, post-partum hemorrhage, and low birth weight) compared to women who were initiated on treatment during the second trimester (incidence of pregnancy complications among women treated during first trimester versus second trimester: 3/31 versus 13/31; *p* = 0.004). None of the other studies in our meta-analysis addressed whether later initiation of levothyroxine had any impact on pregnancy outcomes.

The presence of TPOAb has been shown to increase the risk of pregnancy loss by approximately two-fold among women with SCH [51, 52]. Furthermore, studies have shown that treatment with levothyroxine among women with positive TPOAb levels during pregnancy decreased the rate of pregnancy complications regardless of thyroid function status (i.e. SCH and euthyroid women) [53–55]. In the present meta-analysis, two of the included studies found that levothyroxine treatment in women with SCH and elevated TPOAb was associated with a lower risk of a composite endpoint of gestational hypertension, preeclampsia, anemia, and gestational diabetes [42] and preterm delivery [35]. In contrast, two recent RCTs found that levothyroxine treatment of TPOAb positive women who had normal thyroid function during pregnancy did not affect pregnancy outcomes [56, 57]. However, based on the current evidence, women with SCH and elevated TPOAb may benefit from levothyroxine treatment.

Levothyroxine treatment for SCH during pregnancy may decrease the risk of pregnancy loss among women with infertility [9, 31, 39], although the number of studies in this area is few and the results are conflicting. One study included in our meta-analysis found no association between rates of live births and levothyroxine treatment among women with SCH and recurrent early pregnancy loss [32]. In contrast, the studies by Kim et al. [39] and Al-Anbari [31] found that levothyroxine treatment of SCH among women with infertility, undergoing in vitro fertilization and intracytoplasmic sperm injection, had improved embryo quality and embryo implantation rate compared to women who were not treated. However, the studies were small and further research is needed to determine whether levothyroxine treatment of SCH improves pregnancy outcomes among women with infertility or recurrent pregnancy loss.

### Limitations

Our study has several potential limitations. First, statistical heterogeneity was present in many of our analyses. This heterogeneity is likely due to differences in study design, sample populations, geographical location, and temporal differences in the timing of levothyroxine initiation during pregnancy. There were also some differences in the definition of pregnancy loss across studies, a lack of information relating to TSH levels used to define SCH, and the timing of initiation of levothyroxine therapy. Given this heterogeneity, we used random-effects models to account for the within- and between-study variability. Second, our study defined SCH using the TSH cut-off limit of 2.5mIU/L recommended by the 2011 ATA guidelines [21]. The recent 2017 ATA guidelines recommend various TSH cut-offs based on the presence of thyroid autoimmune disease (i.e. presence of TPOAb) [9] for initiation of levothyroxine treatment. Given that these more recent guidelines heavily rely on the evidence of thyroid autoimmune disease, the use of this definition in our meta-analysis would have resulted in far fewer included studies and could have been affected by selection bias due to the systematic exclusion of older studies. Although the definition of SCH and recommendations for treatment during pregnancy has changed over time, our study provides evidence for treatment of SCH during pregnancy in a more generalized population. Further studies would be required to address the benefits of treating SCH during pregnancy using the recommendations from the 2017 ATA guidelines [9]. Third, outcomes were inconsistently reported, and some of our analyses therefore included a small number of studies. Consequently, some of our estimated treatment effects are accompanied by wide CIs. Due to the small number of studies included in our meta-analysis, our ability to examine the impact of study-level covariates on estimated treatment effects via meta-regression was limited. Fourth, we assessed the association between levothyroxine use and the occurrence of several potential adverse outcomes during pregnancy. The potential for chance findings due to multiple testing should thus be considered when interpreting our findings. Fifth, as is true with all knowledge syntheses, we cannot rule out the possibility of publication bias. Given the small number of included studies, there were an insufficient number of studies to assess publication bias through the use of funnel plots. Sixth, since there is no distribution in the quality of RCTs, we were unable to perform stratified analysis on the quality of these studies. Seventh, the finding of a decreased risk of pregnancy loss associated with levothyroxine treatment versus no treatment was significantly impacted by the findings from Maraka et al. [34] as the other studies are smaller, leading to inconclusive results. Thus, this emphasizes the need to conduct a meta-analysis in this area of research. Eighth, we used a continuity correction of 0.5 to allow for the inclusion of zero-event studies. While this approach reduces potential selection bias, it can introduce bias for small studies with imbalances in numbers. Ninth, the number of studies that addressed the presence of TPOAb was few [35, 42]. We were therefore not able to perform a subgroup analyses among women with SCH secondary to an autoimmune condition. These analyses would have been clinically relevant given that the concurrent presence of an autoimmune disorder may affect fetal outcomes among women with SCH [51, 52]. Tenth, due to the paucity of RCTs in this area of research, we performed meta-analyses of RCTs and observational studies, which may be affected by confounding. However, we conducted stratified analyses to demonstrate findings from RCTs and observational studies separately. Finally, the literature search was conducted on July 18, 2018. Additional studies may have been published since this time.

## Conclusion

This systematic review and meta-analysis found that, compared with non-use, levothyroxine treatment was associated with decreased risks of pregnancy loss and neonatal death among pregnant women with SCH. There was no association between levothyroxine treatment and the risk of other adverse outcomes including outcomes during labour and delivery, and cognitive status in children at 3 or 5 years of age. However, the quality of many of the included studies was modest and important heterogeneity was present. Consequently, further studies are required to address whether levothyroxine treatment among women with SCH improves pregnancy outcomes if given earlier during pregnancy, in women with autoimmune thyroid disease, and in women with a history of infertility or recurrent pregnancy loss.

## Supplementary Information

**Additional file 1.**

## Data Availability

Not applicable.

## Abbreviations

ATA: American Thyroid Association
FT4: Free thyroxine
IQ: Intelligence quotient
PRESS: Peer Review of Electronic Search Strategies
PRISMA: Preferred Reporting Items for Systematic Reviews and Meta-Analyses
PROM: Premature rupture of membranes
RCTs: Randomized controlled trials
ROBINS: Risk of Bias In Non-randomised Studies of Interventions
RR: Risk ratio
SCH: Subclinical hypothyroidism
TPOAb: Anti-thyroid peroxidase antibodies
TSH: Thyroid stimulating hormone
