# Supplementary material for: Levothyroxine and the risk of adverse pregnancy outcomes in women with subclinical hypothyroidism: a systematic review and meta-analysis
Source: BMC Endocr Disord. 2021 Feb 27;21:34. doi: 10.1186/s12902-021-00699-5 (PMC7912520; doi:10.1186/s12902-021-00699-5)
Supplement: Supplementary file 1 — Additional file 1. [file 12902_2021_699_MOESM1_ESM.docx]

**Appendix 1: Ovid Medline Search**

| 1. exp Hypothyroidism/ |  |
| --- | --- |
| 2. Thyroid Diseases/ |  |
| 3. Thyrotropin/ |  |
| 4. Thyroid Gland/ |  |
| 5. Thyroxine/ |  |
| 6. Triiodothyronine/ |  |
| 7. hypo?thyr*.tw,kf. |  |
| 8. ((thyroid* or thyroxin* or tri?odothyronin* or TSH or T4 or T3) adj3 (deficien* or insufficien* or disfunction*)).tw,kf. |  |
| 9. myx?edema*.tw,kf. |  |
| 10. 1 or 2 or 3 or 4 or 5 or 6 or 7 or 8 or 9 |  |
| 11. (mild* or compens* or sub?clinic* or moderat* or short-term or low or antibody negative or asymptomatic).tw,kf. |  |
| 12. ((without or no$1) adj1 (symptom* or manifest*)).tw,kf. |  |
| 13. 11 or 12 |  |
| 14. 10 and 13 |  |
| 15. Pregnancy/ |  |
| 16. Pregnancy in Adolescence/ |  |
| 17. pregnan*.tw,kf. |  |
| 18. 15 or 16 or 17 |  |
| 19. 14 and 18 |  |
| 20. exp Animals/ |  |
| 21. humans.sh. |  |
| 22. 20 not 21 |  |
| 23. 19 not 22 |  |

**Appendix 2: Ovid Embase Search**

| 1. exp hypothyroidism/ |  |
| --- | --- |
| 2. thyroid disease/ |  |
| 3. thyrotropin/ |  |
| 4. thyroid gland/ |  |
| 5. thyroxine/ |  |
| 6. liothyronine/ |  |
| 7. hypo?thyr*.tw,kw. |  |
| 8. ((thyroid* or thyroxin* or tri?odothyronin* or TSH or T4 or T3) adj3 (deficien* or insufficien* or disfunction*)).tw,kw. |  |
| 9. myx?edema*.tw,kw. |  |
| 10. 1 or 2 or 3 or 4 or 5 or 6 or 7 or 8 or 9 |  |
| 11. (mild* or compens* or sub?clinic* or moderat* or short-term or low or antibody negative or asymptomatic).tw,kw. |  |
| 12. ((without or no$1) adj1 (symptom* or manifest*)).tw,kw. |  |
| 13. 11 or 12 |  |
| 14. 10 and 13 |  |
| 15. subclinical hypothyroidism/ |  |
| 16. 14 or 15 |  |
| 17. pregnancy/ |  |
| 18. adolescent pregnancy/ |  |
| 19. pregnan*.tw,kw. |  |
| 20. 17 or 18 or 19 |  |
| 21. 16 and 20 |  |
| 22. exp animal/ |  |
| 23. human.hw. |  |
| 24. 22 not 23 |  |
| 25. 21 not 24 |  |

**Appendix 3: Ebsco Cinahl Plus with Full text Search**

(((((MH "Hypothyroidism+")) OR ((MH "Thyroid Diseases")) OR ((MH "Thyrotropin")) OR ((MH "Thyroid Gland")) OR ((MH "Thyroxine")) OR ((MH "Triiodothyronine")) OR ((TI hypo#thyr*) OR (AB hypo#thyr*) OR (MW hypo#thyr*)) OR ((TI ((thyroid* OR thyroxin* OR tri#odothyronin* OR TSH OR T4 OR T3) N3 (deficien* OR insufficien* OR disfunction*))) OR (AB ((thyroid* OR thyroxin* OR tri#odothyronin* OR TSH OR T4 OR T3) N3 (deficien* OR insufficien* OR disfunction*))) OR (MW ((thyroid* OR thyroxin* OR tri#odothyronin* OR TSH OR T4 OR T3) N3 (deficien* OR insufficien* OR disfunction*)))) OR ((TI myx#edema*) OR (AB myx#edema*) OR (MW myx#edema*))) AND (((TI (mild* OR compens* OR sub#clinic* OR moderat* OR short-term OR low OR antibody negative OR asymptomatic)) OR (AB (mild* OR compens* OR sub#clinic* OR moderat* OR short-term OR low OR antibody negative OR asymptomatic)) OR (MW (mild* OR compens* OR sub#clinic* OR moderat* OR short-term OR low OR antibody negative OR asymptomatic))) OR ((TI ((without OR no OR non "or" "not") N1 (symptom* OR manifest*)) OR MW ((without OR no OR non "or" "not") N1 (symptom* OR manifest*))) OR (AB ((without OR no OR non "or" "not") N1 (symptom* OR manifest*)) OR MW ((without OR no OR non "or" "not") N1 (symptom* OR manifest*))) OR (MW ((without OR no OR non "or" "not") N1 (symptom* OR manifest*)) OR MW ((without OR no OR non "or" "not") N1 (symptom* OR manifest*)))))) AND (((MH "Pregnancy")) OR ((MH "Pregnancy in Adolescence")) OR ((TI pregnan*) OR (AB pregnan*) OR (MW pregnan*)))) NOT (((MH "Animals")) NOT ((MH "Human"))

**Appendix 4: PubMed Search**

((pubmednotmedline[sb] OR publisher[sb]) AND (("pregnancy"[tiab] OR "pregnant"[tiab]) AND (((((((((((("not symptomatic"[tiab] OR "asymptomatic"[tiab]) OR "antibody negative"[tiab]) OR "low"[tiab]) OR "short term"[tiab]) OR "moderately"[tiab]) OR "moderate"[tiab]) OR "subclinical"[tiab]) OR "sub clinical"[tiab]) OR "compensatory"[tiab]) OR "mildly"[tiab]) OR "mild"[tiab]) AND (((((((("disfunctional"[tiab] OR "disfunction"[tiab]) OR "insufficiency"[tiab]) OR "insufficient"[tiab]) OR "deficiency"[tiab]) OR "deficient"[tiab]) AND ((((((((("t3"[tiab] OR "t4"[tiab]) OR "tsh"[tiab]) OR "triiodothyronine"[tiab]) OR "triiodothyronin"[tiab]) OR "triodothyronin"[tiab]) OR "triodothyronine"[tiab]) OR "thyroxine"[tiab]) OR "thyroxin"[tiab]) OR "thyroid"[tiab])) OR "hypothyroidism"[tiab]) OR "hypothyroid"[tiab])))) NOT ("animals"[mesh] NOT "humans"[mesh])

**Supplementary Table 1: Quality assessment of prospective trials**

| **Study** | **Random-ization** | **Allocation concealment** | **Blinding participants**  **& personnel** | **Blinding outcomes** | **Attrition bias** | **Reporting bias** | **Other bias** |
| --- | --- | --- | --- | --- | --- | --- | --- |
| Kim et al. 2011 | Yes | No† | No † | No † | No | No | * |
| Lazarus et al. 2012 | Yes | No | No | Yes | Yes | No |  |
| Al-Anbari 2017 | No | No | No | No | No | No |  |
| Casey et al. 2017 | Yes | Yes | Yes | Yes | No | No |  |
| Nazarpour et al. 2017 | Yes | Yes | No | Yes | Yes | Yes | * |
| Nazarpour et al. 2018 | Yes | Yes | No | Yes | No | Yes § | * |
| Zhao et al. 2018 | Yes | No | No | No | No | No |  |

† No blinding protocol declared and the allocation sequence was provided to personnel

* Multiple comparisons

§ Subgroup sample size not reported. Heterogenous starting gestational age for levothyroxine replacement therapy with conflicting report in text

**Supplementary Table 2: Quality assessment of cohort studies using the ROBINS I framework**

| **Study** | **Confounds** | **Selection** | **Interven-tion classif-ication** | **Interven-tion deviation** | **Missing data** | **Outcome measure-ment** | **Result Selection** | **Overall** |
| --- | --- | --- | --- | --- | --- | --- | --- | --- |
| Bernardi et al. 2013 | Moderate | low | low | low | low | low | low | low |
| Wang et al. 2012 | serious | moderate | low | low | low | low | low | serious |
| Maraka et al. 2016 | moderate | moderate | low | low | low | low | moderate | moderate |
| Ju et al. 2016 | critical | serious | low | low | low | moderate | serious | serious |
| Zhang et al. 2017 | critical | low | moderate | low | low | moderate | moderate | critical |
| Maraka et al. 2017 | moderate | moderate | low | low | low | low | moderate | moderate |

| **Outcomes** | **0.1 Continuity Correction** | | **0.5 Continuity Correction** | |
| --- | --- | --- | --- | --- |
|  | **Risk ratio** | **95% confidence interval** | **Risk ratio** | **95% confidence interval** |
| APGAR under 7 at 5 minutes | 0.42 | 0.03-5.34 | 0.46 | 0.02-8.84 |
| Intrauterine growth restriction | 1.06 | 0.59-1.90 | 1.06 | 0.59-1.90 |
| Low birth weight | 0.80 | 0.26-2.43 | 0.76 | 0.23-2.54 |
| Neonatal death | 0.35 | 0.17-0.72 | 0.09 | 0.05-0.16 |
| Placental abruption | 0.98 | 0.37-2.61 | 0.89 | 0.34-2.33 |
| Postpartum hemorrhage | 0.88 | 0.18-4.22 | 0.84 | 0.16-4.40 |
| Pregnancy loss | 0.79 | 0.67-0.95 | 0.80 | 0.68-0.93 |
| Preterm delivery | 0.77 | 0.47-1.25 | 0.77 | 0.47-1.27 |
| Preterm labour | 1.05 | 0.56-1.97 | 1.05 | 0.56-1.97 |
| Premature rupture of membranes | 0.94 | 0.52-1.70 | 0.94 | 0.52-1.70 |
| Spontaneous abortion | 0.46 | 0.04-5.32 | 0.54 | 0.06-4.96 |
| Still birth | 0.90 | 0.54-1.51 | 0.90 | 0.53-1.52 |

**Supplementary Table 3: Sensitivity analysis using a continuity correction of 0.5 and 0.1 in the meta-analyses assessing the association between levithyroxine treatment versus no treatment and the risk of adverse outcomes during oregnancy among women with subclinical hypothyroidism.**

**Supplementary Figure 1 (S1): Random-effects meta-analysis of intrauterine growth restriction associated with levothyroxine treatment versus no treatment among women with subclinical hypothyroidism during pregnancy presented as a risk ratio (RR) and 95% confidence interval (CI)**

**Supplementary Figure 2 (S2): Random-effects meta-analysis of spontaneous abortion associated with levothyroxine treatment versus no treatment among women with subclinical hypothyroidism during pregnancy presented as a risk ratio (RR) and 95% confidence interval (CI)**

**Supplementary Figure 3 (S3): Random-effects meta-analysis of placental abruption associated with levothyroxine treatment versus no treatment among women with subclinical hypothyroidism during pregnancy presented as a risk ratio (RR) and 95% confidence interval (CI)**

**Supplementary Figure 4 (S4): Random-effects meta-analysis of postpartum hemorrhage associated with levothyroxine treatment versus no treatment among women with subclinical hypothyroidism during pregnancy presented as a risk ratio (RR) and 95% confidence interval (CI)**

**Supplementary Figure 5 (S5): Random-effects meta-analysis of premature rupture of membranes associated with levothyroxine treatment versus no treatment among women with subclinical hypothyroidism during pregnancy presented as a risk ratio (RR) and 95% confidence interval (CI)**

**Supplementary Figure 6 (S6): Random-effects meta-analysis of preterm delivery associated with levothyroxine treatment versus no treatment among women with subclinical hypothyroidism during pregnancy presented as a risk ratio (RR) and 95% confidence interval (CI)**

**Supplementary Figure 7 (S7): Random-effects meta-analysis of preterm labor associated with levothyroxine treatment versus no treatment among women with subclinical hypothyroidism during pregnancy presented as a risk ratio (RR) and 95% confidence interval (CI)**

**Supplementary Figure 8 (S8): Random-effects meta-analysis of neonatal death associated with levothyroxine treatment versus no treatment among women with subclinical hypothyroidism during pregnancy presented as a risk ratio (RR) and 95% confidence interval (CI)**

**Supplementary Figure 9 (S9): Random-effects meta-analysis of low Apgar score (less than 7 at 5 minutes) associated with levothyroxine treatment versus no treatment among women with subclinical hypothyroidism during pregnancy presented as a risk ratio (RR) and 95% confidence interval (CI)**

**Supplementary Figure 10 (S10): Random-effects meta-analysis of low birth weight associated with levothyroxine treatment versus no treatment among women with subclinical hypothyroidism during pregnancy presented as a risk ratio (RR) and 95% confidence interval (CI)**

**Supplementary Figure 11 (S11): Random-effects meta-analysis of pregnancy loss associated with levothyroxine treatment versus no treatment among women with subclinical hypothyroidism during pregnancy and no known history of infertility or recurrent pregnancy loss presented as a risk ratio (RR) and 95% confidence interval (CI)**

**Supplementary Figure 12 (S12): Random-effects meta-analysis of pregnancy loss associated with levothyroxine treatment versus no treatment among women with subclinical hypothyroidism during pregnancy after exclusion of the study by Al-Anbari 2017**

**Supplementary Figure 13 (S13): Random-effects meta-analysis of pregnancy loss associated with levothyroxine treatment versus no treatment among women with subclinical hypothyroidism during pregnancy in observational studies stratified by quality of study (studies with high risk of bias versus studies with moderate risk of bias) presented as a risk ratio (RR) and 95% confidence interval (CI).**

**Supplementary Figure S14 (S14): Influence analysis assessing the risk of pregnancy loss associated with levothyroxine treatment versus no treatment among women with subclinical hypothyroidism during pregnancy.**
